# Adult Stem Cells in Aging

**DOI:** 10.3390/jpm12050795

**Published:** 2022-05-14

**Authors:** Drenka Trivanović

**Affiliations:** 1 IZKF Group Tissue Regeneration in Musculoskeletal Diseases, University Hospital Wuerzburg, 97070 Wuerzburg, Germany; drenka.trivanovic@imi.bg.ac.rs; 2 Bernhard-Heine-Center for Locomotion Research, University of Wuerzburg, 97070 Wuerzburg, Germany; 3 Group for Hematology and Stem Cells, Institute for Medical Research, National Institute of Republic of Serbia, University of Belgrade, 11000 Belgrade, Serbia

Aging process is associated with numerous intrinsic and extrinsic factors that contribute to the adipose tissue accumulation, atherosclerosis, immune system failures, bone fragility, and cancer. The aforementioned pathophysiological states represent critical threats to modern society, and it is of paramount importance to define the “senescent” cell phenotype and to depict possible targets to prevent an undesirable aging process. With aging, the stem cell quiescence period is disturbed, which leads to their uncontrolled activation and their potential exhaustion. At the cellular level, senescence leads to alternated metabolic phenotype or oncogenic events. Both paths involve the aberrant bioenergetics, cytoskeleton, mitochondrial function and cell signalling, directly affecting cell redox homeostasis, divisions, and cell death program [1,2]. Thus, changes provoked by reactive oxygen species, the dysregulation of autophagy, and altered epigenetic landscapes are associated with aging in stem cells. Several studies have specifically focused on reprogramming aged stem cells into induced pluripotent stem cells and then re-differentiating them back into the original stem cell with restored potential. Moreover, diet-based approaches (caloric restriction, fasting), extracellular component modification, or metabolite supplementation (NAD+ level restore, mTOR inhibition, senolytics) are promising, so-called “rejuvenating” strategies for delay of stem cell aging [3]. This Special Issue brings several novel findings and the most recent literature overviews on mechanisms of adult stem cell aging and strategies to combat it.

The aging process in mesenchymal stromal/stem cells (MSC) is under intensive investigations, where the context-dependent behavior of MSC in the aged microenvironment is of great importance [4]. Authors recognized that the youthfulness of MSCs seems to be essential for the success of the therapy. They also considered that differences between in vivo-aged cells, coming from elderly donors, or in vitro-aged cells, derived from late culture passages, show a senescent phenotype with a decline in quality, being key in the development of age-related pathologies. Through a literature overview of MSCs’ role in aging-related diseases and their rejuvenating potential, evidences summarized in this review point that cell-based or cell-free therapies based on young MSCs should be considered as realistic interventions to counteract aging. Similarly, another review paper [5] summarized current knowledge on potential usefulness of adipose tissue stromal cells in rejuvenation strategies and significance of estimation of their age status prior to their application.

The interaction between the circadian rhythms of gene expression and epigenetic clocks characterized by the specific DNA methylation in CpG-islands which are associated with the senescence of all somatic and stem cells have also been described [6]. Namely, that mechanisms of epigenetic regulation of the circadian rhythms of transcription and their role in the regulation of the cell cycle during aging are currently being actively studied. The authors described the aging process in hematopoietic stem cells, MSCs, intestinal stem cells, satellite stem cells, neural stem cells, skin stem cells, and germinal stem cells. Epigenetic clocks are sensitive enough to detect even minor changes in the biological age resulting from longevity and reprogramming interventions. Thus, reprogramming without reaching the pluripotent stage may be viewed as a way of epigenetic rejuvenation of cells and tissues.

Adult bone marrow MSCs were extensively studied for their ability to support hematopoietic process in mice and humans and were recognized as important and reactive components of the HSC niche. Moreover, chemotherapy is known as an inducer of changes in both hematopoietic compartments and MSC functions. The authors investigated how hydroxyurea, an inhibitor of ribonucleotide reductase, alter human MSC differentiation and immunosuppressive functions [7]. This study showed that hydroxyurea induced MSC senescence including cell proliferation arrest, which suggests a potential “therapy-induced senescence” effect. The aforementioned events were followed by impaired cell differentiation and increased immunosuppressive activity of immune T-cells.

Although recognized, the connection between MSC senescence and cancer development has not been completely explored. Numerous features of aging MSCs, including altered immunomodulatory properties, impeded MSC-niche-supporting functions, and the senescent MSC secretory repertoire are consistent with inflammation development [8]. The article described the tumor-promoting roles of senescent MSC and the therapeutic relevance of targeting senescent MSCs. In addition, another review paper discussed the possibility that MSCs can achieve properties of senescent cells in acute myeloid leukemia and B-acute lymphoblastic leukemia. It appears that these different malignancies can induce similar senescent profiles in MSCs as observed in aged bone marrow, particularly in terms of redox status and epigenetics [9].

Taken together, I hope that this Special Issue provides interesting insights on the complexity of adult stem cell responses to the aging process and supports further development of anti-aging strategies. I also believe that this Special Issue raises new questions which will be explored and clarified in future studies.
